# Emotional recognition for simulated clinical environment using unpleasant odors: quasi-experimental study*

**DOI:** 10.1590/1518-8345.2883.3248

**Published:** 2020-02-14

**Authors:** Mateus Henrique Gonçalves Meska, Leandro Yukio Mano, Janaina Pereira Silva, Gerson Alves Pereira, Alessandra Mazzo

**Affiliations:** 1 Universidade de São Paulo, Escola de Enfermagem de Ribeirão Preto, Collaborating Centre For Nursing Research Development, Ribeirão Preto, SP, Brazil.; 2 Scholarship holder at the Fundação de Amparo à Pesquisa do Estado de São Paulo (FAPESP), Brazil.; 3 Universidade de São Paulo, Instituto de Ciências Matemáticas e Computação, São Carlos, SP, Brazil.; 4 Universidade de São Paulo, Faculdade de Odontologia, Bauru, SP, Brazil.; 5 Scholarship holder at the Conselho Nacional de Desenvolvimento Científico e Tecnológico (CNPq), Brazil.

**Keywords:** Simulation, Patient Simulation, Odorants, Education, Education, Nursing, Education, Higher, Simulação, Simulação de Paciente, Odorantes, Educação, Educação em Enfermagem, Educação Superior, Simulación, Simulación de Paciente, Odorantes, Educación, Educación en Enfermería, Educación Superior

## Abstract

**Objective:**

to compare the effect of exposure to unpleasant odors in a simulated clinical environment on the emotions of undergraduate nursing students.

**Method:**

quasi-experimental study. A total of 24 nursing students participated the study, divided into two groups, 12 in the intervention group with exposure to unpleasant odors, and 12 in the control group without exposure to unpleasant odors. To simulate the unpleasant vomiting odor in intervention group, fermented foods were used: boiled oats, curdled milk, spoiled Parmesan cheese, raw egg, pea soup, raisins and vinegar. Participants were filmed and the facial expression analysis was performed at six critical points: student approach; report of the complaint; clinical evaluation; and patient occurrence, intervention and reevaluation based on what was proposed by the Circumplex model of emotions recognition.

**Results:**

a total of 83,215 emotions related to the six critical points were verified. At the critical point of the proposed scenario with exposure to unpleasant odors, the intervention group presented the basic emotion of sadness and the Control Group, anger.

**Conclusion:**

it is inferred that the inclusion of unpleasant odors in the simulated scenarios can broaden the emotional development of health students.

## Abstract (pt)

**Objetivo:**

comparar o efeito da exposição aos odores desagradáveis em ambiente clínico simulado nas emoções de estudantes de graduação de enfermagem.

**Método:**

estudo quase experimental. Participaram 24 estudantes de enfermagem, alocados em dois grupos, 12 no Grupo Intervenção com exposição aos odores desagradáveis e 12 no Grupo Controle sem exposição aos odores desagradáveis. Para simulação do odor desagradável de vômito, no Grupo Intervenção, foram utilizados alimentos fermentados: aveia cozida, leite coalhado, queijo parmesão estragado, ovo cru, sopa ervilha, uvas-passas e vinagre. Os participantes foram filmados e a análise das expressões faciais foi realizada em seis pontos críticos: abordagem pelo estudante; relato da queixa; avaliação clínica; e intercorrência, intervenção e reavaliação do paciente a partir do proposto pelo modelo *Circumplex* de reconhecimento de emoções.

**Resultados:**

verificou-se 83.215 emoções relacionadas aos seis pontos críticos. No ponto crítico do cenário proposto com exposição aos odores desagradáveis o grupo intervenção apresentou a emoção básica de tristeza e o grupo controle, raiva.

**Conclusão:**

infere-se que a inclusão de odores desagradáveis nos cenários simulados pode ampliar o desenvolvimento emocional de estudantes da área da saúde.

## Abstract (es)

**Objetivo:**

comparar el efecto de la exposición a los olores desagradables en ambiente clínico simulado en las emociones de estudiantes de grado de enfermería.

**Método:**

estudio cuasiexperimental. Participaron 24 estudiantes de enfermería, distribuidos en dos grupos, 12 en el Grupo Intervención con exposición a los olores desagradables y 12 en el Grupo Control sin exposición a los olores desagradables. Para simular el olor desagradable de vómito, en el grupo intervención, se utilizaron alimentos fermentados: avena cocida, leche cuajada, queso parmesano estropeado, huevo crudo, sopa de arvejas, uvas pasas y vinagre. Los participantes fueron filmados y las expresiones faciales se analizaron en relación con seis puntos críticos: abordaje por el estudiante; relato de la queja; evaluación clínica; e intercurrencia, intervención y reevaluación del paciente con base en lo propuesto por el modelo *Circumplex* de reconocimiento de emociones.

**Resultados:**

se verificaron 83.215 emociones relacionadas con los seis pontos críticos. En el punto crítico del escenario propuesto con exposición a los olores desagradables, el grupo intervención presentó la emoción básica tristeza; y el grupo control, rabia.

**Conclusión:**

se deduce que la inclusión de olores desagradables en los escenarios simulados puede ampliar el desarrollo emocional de estudiantes del área de la salud.

## Introduction

Simulation is recognized as an effective method in the teaching and learning process in the training of health professionals. Besides improvement of skills, it includes the use of clinical scenarios that recreate real situations of professional life in safe and controlled environments. When systematically developed, simulation brings positive results to learners^(^ 1 ^-^ 2 ^)^.

Clinical simulation can also be used to investigate and/or improve other factors in student education, such as psychomotor, cognitive and affective aspects, such as verbal and nonverbal communication skills, teamwork, interprofessional work, interpersonal relationships^(^ 1 ^)^, behaviors and emotions^(^ 3 ^-^ 4 ^)^.

In this context, clinical simulation enables students to be prepared to experience situations in real contexts of the profession and favors the development of attitudinal aspects that are inherent to the profession, such as verbal and nonverbal communication^(^ 5 ^)^. Nonverbal expression is a critical point in the communication process, since the control of reactions by students to patients is not always adequate, which may compromise the quality of care. Unpleasant odors are a very common situation in nurses’ daily lives, which can affect nonverbal communication with the patient^(^ 5 ^-^ 7 ^)^.

When faced with unpleasant odors, professionals and/or students may express nonverbal signals that can be noticed, contextualized, interpreted and judged by the patient^(^ 4 ^,^ 8 ^)^. Although professionals and students try to disguise the awkward situation, nonverbal people speak louder and are easily understood by the patient.

To minimize the contradictory effects of nonverbal reactions, it is important to understand students’ emotional aspects in situations involving unpleasant odors.

Emotions are subjective reactions to a particular environmental event, internal or external, and are characterized by physiological, cognitive and behavioral changes that allow the individual to attribute meaning to the experience and prepare him/her for a particular action^(^ 9 ^)^. Emotions are adaptive because they provide, predispose and guide behaviors, and provide information about problem situations in which individuals are involved^(^ 9 ^-^ 11 ^)^.

The known basic emotions are joy, aversion, fear, neutral, anger, surprise and sadness^(^ 12 ^-^ 13 ^)^, in addition to the neutral state, also considered and used as a reference of emotional states. In this sense, the representations of emotions have been used in several computational applications with good performance^(^ 11 ^,^ 14 ^-^ 15 ^)^.

The wide spectrum of applications and the ever-increasing computational processing power have motivated researchers to identify user emotions in many business and research contexts, and also to use this information as a basis, for example, for decision making, satisfaction analysis and behavior in the execution of tasks^(^ 15 ^)^. In fact, classification procedures have helped in the analysis of emotional responses, aiding in the diagnosis of depression, behavior change, among others, and thus providing the opportunity for emotional analysis also in simulated environments^(^ 16 ^)^.

Emotions, in their broadest aspects, may semantically be equal to facial and gesture expressions, which are subjectively experienced^(^ 9 ^-^ 10 ^)^. When we reflect on an action, we experience emotional reactions based on our expectation about the solutions we have given them in past experiences, and thereby regulate our future behaviors. There is then a close connection between emotion, cognition and motivation^(^ 9 ^)^.

In order not to expose the patient, emotions can be experienced and reflected in simulated practices. Well-designed clinical scenarios add truth to experiences that would only be experienced in real practice. In this context, this study aims to compare the effect of exposure to unpleasant odors in simulated clinical environment on the emotions of undergraduate nursing students.

## Method

This is a quasi-experimental study^(^ 17 ^)^, approved by the Research Ethics Committee of the University of São Paulo at Ribeirão Preto College of Nursing under Opinion No. 322/2016. The acceptance to participate in the study was formalized by signing the Informed Consent Form; there were no refusals.

The eligibility criteria were undergraduate nursing students, aged 18 and over, from public or private educational institutions, enrolled in any semester, with or without experience in simulated practices and clinical practices in teaching internships.

A total of 24 nursing students who met the eligibility criteria were part of the study and were randomly allocated to two groups, Intervention Group (IG) and Control Group (CG). Students allocated in the IG (n=12) participated in simulated scenarios with the presence of unpleasant odors, and students allocated in the CG (n=12) in simulated scenarios without those odors.

The study was conducted at a public university in the countryside of the state of São Paulo. To accomplish this, two days of a simulated workshop were offered to undergraduate nursing students. The event was publicized online, on the website of the institution where it was held and its central theme was “Nursing care for hospitalized clinical patients.” Registration was available free of charge and participants could only register for one day of the workshop offered. All participants received material for prior reading on the topics to be addressed at the event. During classroom activities, students were invited to participate in the study.

The event was held on two separate days to different audiences, previously registered, and lasted four hours a day. During the event, undergraduates participated in four distinct simulated clinical scenarios, of medium and high fidelity, and their respective *debriefings*.

Each scenario had a central theme: nursing care for patients with vomiting for gastric disorders; nursing care for adult patients with intestinal elimination in disposable diaper; nursing care for patients with infected skin lesions; and nursing care for colostomy patients.

The scenario entitled “Nursing care for patients with vomiting due to gastric disorders” was chosen by the researchers. On the first day, the CG participated, and on the second day the same scenario was offered to the IG, with the addition of unpleasant odors. The same scenario was led by the same facilitator on both days.

To simulate the unpleasant vomiting odor in IG, fermented foods were used: boiled oats, curdled milk, spoiled Parmesan cheese, raw egg, pea soup, raisins and vinegar. The scenario was elaborated based on literature review and expert opinion. Constructed from a script^(^ 18 ^)^, the scenarios were validated in appearance and content by a group of five experts. There was 100% agreement between the judges^(^ 19 ^)^.

All enrolled students participated in the simulated practices. In scenario 1, two students acted as volunteers and the others were observers, following the recommendation of the judges of the content validation stage. The participants had 5 minutes to recognize the environment with their pre*-*briefing and briefing, 20 minutes to develop the scenario, and 30 minutes to conduct their structured debriefing. 

For data collection during the development of the analyzed scenario, audio/video cameras were arranged to record the students’ performance in the development of the scenario. The cameras were placed on a tripod positioned on the left and right sides of the headboard, consequently recording the faces of the two participants. Such footage was directed to the analysis of students’ facial expressions during patient care to determine the emotion presented in the groups that witnessed and did not witness unpleasant odors, and the classification of the student’s face for emotion analysis was divided into three stages:

- Face Detection Automatically finds the face region. This stage can be influenced by head movement, lighting, presence of hair and glasses.
- Extraction of facial features: step based on geometric features. Methods based on geometric features are used in facial modeling, i.e. motor expressions, to develop a similar approach to the way humans interpret the elements of the face. Different facial representations, such as joy, fear, neutral, anger, surprise and sadness are proposed for the identification and classification of emotions, being able to encode an individual’s facial configuration^(^ 12 ^)^. In this study we used a software^(^ 14 ^)^, which is characterized as a computer vision system to obtain facial information. In it, 33 facial points are used: 8 mapped points of the mouth, 6 for each eye, 3 for each eyebrow and chin, 2 for the nostrils and 2 delimiting the lateral ends of the face. Figure 1 presents an example of the face mapping performed and used in this study.
  **Figure 1:**
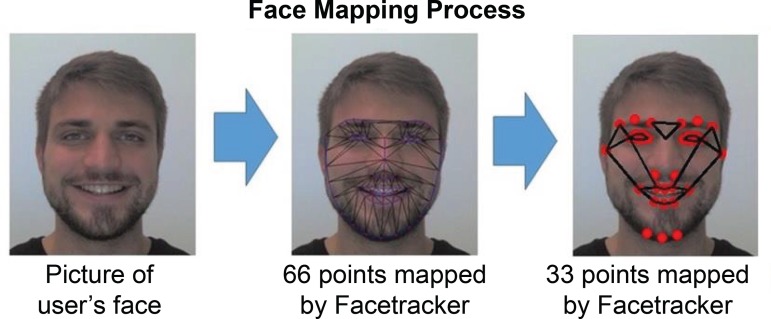
Face mapping process^(^ 14 ^)^

Source: copyright of the author
- Face Classification: it was performed through a set of *Machine Learning* based algorithms from a facial reference model composed of 33 characteristic points. The algorithm seeks to align the elements of the face under analysis with the characteristic points of the reference model^(^ 14 ^)^.

It is noteworthy that the software for facial recognition of emotions is based on artificial intelligence algorithms and is based on the Classifiers Committee approach, that is, the combination of classifiers is intended to lead to a performance improvement in terms of generalization and increased classification accuracy^(^ 14 ^)^. The model used in this study has been tested and validated in previous studies^(^ 14 ^-^ 15 ^,^ 20 ^)^, achieving an average accuracy of 82.53% in the classification of emotion by face, and provides evidence of emotion expressed by the individual. Moreover, this emotion recognition system has been used in previous studies^(^ 16 ^,^ 21 ^)^ to evaluate students in clinical simulation, providing a different perspective regarding student analysis in simulated scenarios.

Categorical representation, that is, facial emotional analysis and the set of related facial expressions, implies changes in the face that accompany the user’s emotional experience^(^ 12 ^)^. Within this context, the face undergoes changes according to the degree of excitement; in terms of emotional responses you have, for example, a look of hatred, frowning, pinching lips, or even a smile. All other emotional categories are then constructed from combinations of the basic emotions, such as joy, aversion, fear, anger, surprise and sadness^(^ 12 ^)^. It is noted that the main advantage of a representation through categorical schema is the similarity in how people use such schema to describe emotional demonstrations observed in everyday life.

**Figure 2:**
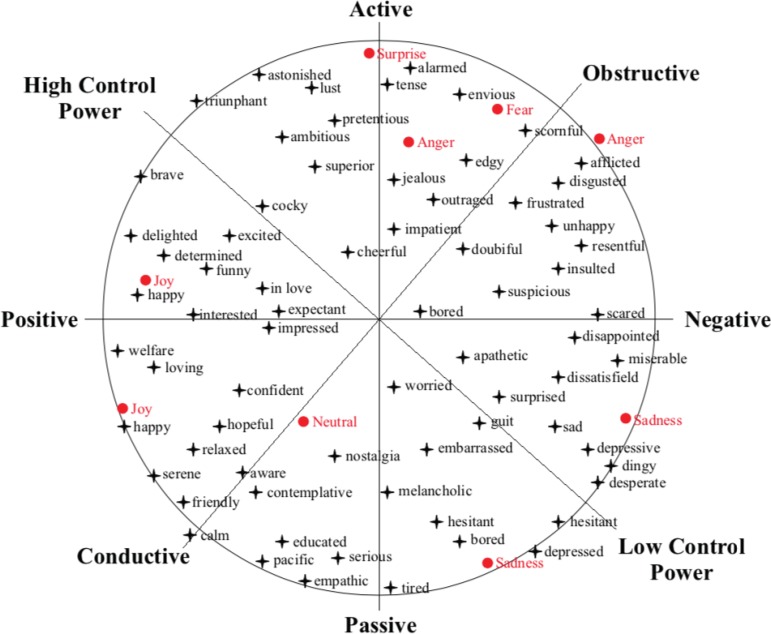
Circumplex Model^(^ 11 ^)^

For the students’ emotional analysis, the Circumplex model^(^ 11 ^))^ (Figure 2) was used as a dimensional approach that argues that all basic emotions are in a continuous two-dimensional space, where the dimensions are: “valence”, that corresponds to the type of emotion and represent how a human being feels (X axis - positive or negative); “Excitement”, corresponds to the intensity of emotion and measures the propensity of human beings to perform an action triggered by the emotional state (Y axis - active or passive, linked to the level of energy or excitement associated with the emotion); *coping potential* (main diagonal), assesses the organism’s sense of control, high or low, over a given event; and *goal attainment* (secondary diagonal - conductive or obstructive, to assess the ease of achieving one or more objectives).

Footage for facial expression analysis was analyzed individually by the emotional recognition software based on six critical points of the scenario identified by the researchers: student approach to the patient; report of the patient’s complaint; clinical assessment of the patient; occurrence - patient vomiting, moment when the student kept contact with unpleasant odor; intervention; and patient reevaluation. This division allowed us to verify, based on the classification of emotions and the relationship with the Circumplex model^(^ 11 ^)^, both the most latent emotions and the time applied at each moment of the proposed scenario.

## Results

Among the undergraduate nursing students, 23 (95.8%) were female and 1 (4.2%) male. Of the participants, 2 (8.3%) were in the second year, 10 (41.7%) the third and 10 (41.7%) the fourth year. All students had participated in practical laboratory training and 20 (83.3%) in simulated practices. No student had participated in a simulated scenario using unpleasant odors.

**Figure 3:**
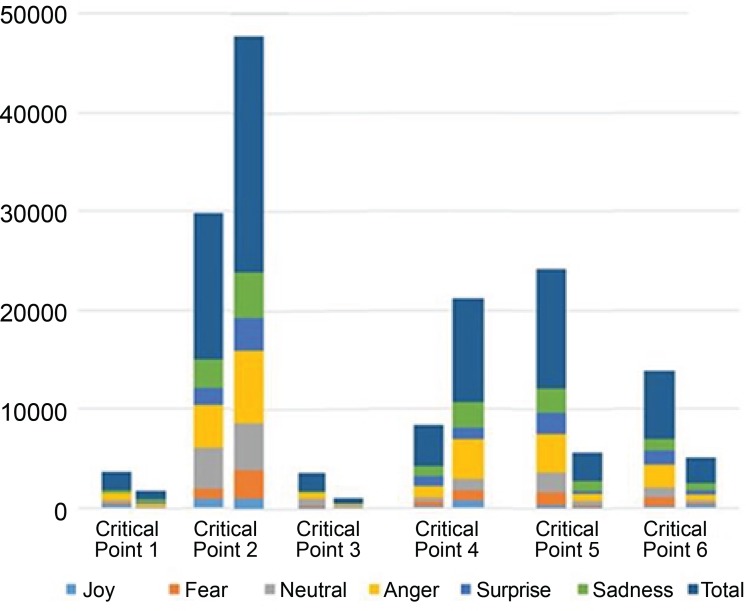
Number of emotions in the six critical points of the Control Group and Intervention Group scenario. Ribeirão Preto, SP, Brazil, 2018

During the simulated scenario, based on the six critical points, 83,215 emotions were identified through the emotional recognition software. Figure 3 shows the total number in each group and at each critical point in the scenario. Table 1 presents the results (in percentage) of the emotions experienced in the proposed experiment.

**Table 1:** Distribution of the six critical points of the scenarios in the Control Group and Intervention Group, according to the classification of emotions in percentage (%). Ribeirão Preto, SP, Brazil, 2018

	Joy	Fear	Neutral	Anger	Surprise	Sadness
Student Approach by the Patient						
*Control Group*	19.2	3.8	23.3	33.0	6.1	14.3
*Intervention Group*	8.1	5.6	14.2	32.5	13.8	25.5
Reporting of the patient's complaint						
*Control Group*	5.9	7.4	27.3	29.3	10.9	19.0
*Intervention Group*	4.5	12.1	19.3	30.8	13.7	19.2
Clinical assessment of the patient						
*Control Group*	11.4	3.5	46.5	24.9	3.5	10.0
*Intervention Group*	12.0	5.5	46.8	15.6	8.4	11.4
Occurrence - Patient Vomiting						
*Control Group*	6.4	8.8	10.2	27.5	22.8	24.1
*Intervention Group*	6.6	9.8	10.5	38.8	10.2	23.8
Intervention						
*Control Group*	3.2	9.8	17.5	32.1	17.8	19.3
*Intervention Group*	7.8	5.1	12.1	27.1	9.8	37.8
Reevaluation						
*Control Group*	3.6	10.8	15.4	33.4	20.6	15.9
*Intervention Group*	14.1	4.9	12.7	23.1	17.4	27.6

**Figure 4:**
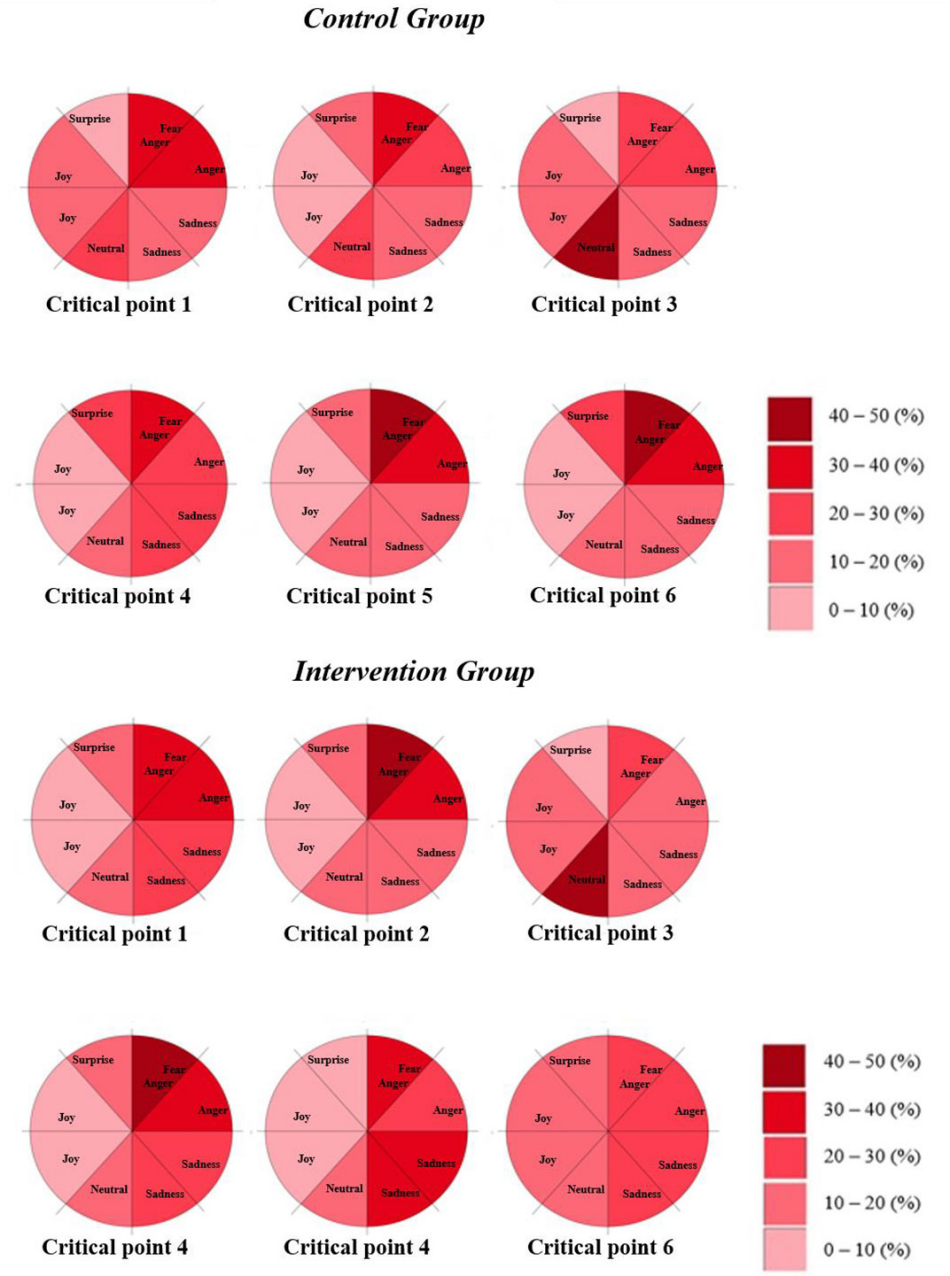
Critical points defined in the scenario of the Control and Intervention groups, according to the Circumplex model^(^ 11 ^)^, in percentage. Ribeirão Preto, SP, Brazil, 2018

Based on the percentage analysis of the groups, Figure 4 shows the emotions according to the Circumplex model of the six critical points of the scenario.

## Discussion

Emotion organizes individual components, feelings, activation, purpose, and expressions into a coherent reaction to a provocative event^(^ 22 ^-^ 23 ^)^. The results of this study showed that CG and IG students predominantly presented the emotion of “anger”. After the presence of unpleasant odors, the emotion “sadness” in the IG predominates.

Thus, the use of computational resources to recognize emotions in simulated activities, as well as material resources that bring greater truth to the simulation, such as the incorporation of unpleasant odors in simulated practices, help the teaching and learning process, and contribute positively to learner development. In addition, when the simulated clinical scenarios are structured with clear objectives, they can promote improvement in student interaction in their training process, also bringing a self-reflective process on the care provided.

When analyzing Figure 4, it is observed that at moments 1, 2 and 4 of the critical points of the scenario both groups showed the emotion “anger”. Anger can be interpreted as a negative valence degree, with a positive excitement-related energy level, with a high potential for coping and control, and with a degree of obstructive range with respect to assessing the ease of achieving the desired goals and outcomes related to feelings such as tension, alarm, irritation, impatience, doubt and distrust^(^ 11 ^)^.

Anger is the most passionate emotion and arises from restraint, such as when one’s plans or well-being are influenced by some external force. The angry person has more energy, increases people’s sense of control, becomes more sensitive and perceptive. Anger creates a motivational desire to do what, if it were not for it, the event might not be fulfilled^(^ 24 ^-^ 26 ^)^.

The presence of ‘angry’ emotion among students is not harmful to the teaching and learning process during simulated scenarios. Such a feeling adapts the body to stress reactions. Studies^(^ 16 ^,^ 21 ^,^ 27 ^)^ show a relationship between stress level and student learning. When stressed, students develop a model of bodily stress responses that consists of three stages: alarm, physiological excitement, and defense. During the alarm, the organism perceives the stressor and mobilizes; in the physiological excitement of the body, it concentrates the resources to meet the challenge; and, finally, in defense, the organism manifests resistance and exhaustion^(^ 27 ^)^.

Learning is the result of a complex process involving the activation of specific neural networks as a result of the environmental stimuli presented. Emotional factors exert a strong influence on this process and should be considered by educators and education managers^(^ 28 ^)^. In this sense, it is observed that the emotion “anger” is an important factor in the teaching and learning process and the results showed that the use of simulated clinical scenarios is a strategy that allows the awakening of motivating emotions of learning.

The moment 3 “Patient Assessment”, as shown in Table 1 and Figure 4, showed the “neutral” emotion in both groups. Neutral refers to a degree of positive valence and a level of energy or excitement associated with passive emotion, with low control and coping power, and a degree of conductive range to assess the ease of achieving goals and outcomes. Neutral feeling can also be linked to emotions such as serious, attentive, polite, peaceful and empathetic^(^ 11 ^)^. Patient assessment is the moment when the student needs to be alerted to look for signs and symptoms for decision making. Thus, for teaching and learning strategies that focus on clinical assessment, the simulated skill training practice is more accurate for students.

After point 4 of the scenario, “patient vomiting”, at critical points 5 and 6, the CG remained with the predominance “anger”. However, the IG, when faced with the unpleasant smell of vomiting, began to have as predominant emotion “sadness” at critical points 5 and 6, as indicated in Table 1. The sadness emotion is related to the emotions of depression, shame, worry, surprise, dissatisfaction, disappointment, which refer to the degree of negative valence, with passive excitement level, control power and low coping and conductive range to assess the ease to achieve goals^(^ 11 ^)^.

Sadness, because of unpleasant feeling, motivates the individual to take on behaviors necessary to soften circumstances that promote distress before they occur again. In addition, it can motivate the individual to return to the previous state of a distressing situation. Although sadness makes the person feel unhappy, it can also maintain productive behaviors, because the student is more motivated to be prepared and to avoid the possibility of suffering the anguish that led to sadness^(^ 24 ^-^ 25 ^,^ 29 ^)^. In this sense, when experiencing unpleasant odors in simulated clinical environments, students can reflect on coping with this situation along with the patient in real clinical practice, working their nonverbal communication, which will minimize the embarrassment of patients and future professionals.

In this approach, the students, when experiencing simulated clinical scenarios, experience emotions that direct attention and channel behaviors, according to the circumstances faced. Each emotion provides unique readiness to respond to a particular situation; they are therefore positive, functional, purposeful and adaptive organizers of behavior^(^ 24 ^-^ 25 ^,^ 29 ^)^. Emotions have a strong influence on the learning process, and their understanding in the educational context is relevant. Through emotions, students expose to educators the characteristics of their personality, their difficulties or skills, which are still developing throughout their formation^(^ 22 ^)^.

Expressions of emotions are powerful nonverbal messages that communicate to others the feelings they experience, influence how people interact, and can promote behavioral reactions in the other person. Although much of the facial expression component of emotions is timely learned during human development and work and is voluntary behavior, the possibility of facial behavior having an innate genetic component is not eliminated. A series of research across cultures has tested the proposition that humans show similar facial expressions regardless of cultural differences^(^ 12 ^,^ 15 ^,^ 24 ^-^ 25 ^)^, which may be one of the limiting factors of this study.

The teaching and learning process goes beyond the simple knowledge acquisition, and it is the trainers’ job to know all the resources that can be associated with this purpose to recognize, analyze, select and apply the best strategies, ensuring the formation of professionals more prepared to deal with clinical practice, while providing the student a fruitful environment for discovery and learning.

Emotions play an important role in the construction of meanings in the teaching and learning process, related to the impulses, interests and motivations of students and the trainer in the knowledge acquisition^(^ 19 ^)^. In this sense, it is essential for the trainer to make it a support tool in teaching strategies, not only for the development of skills, but also to enable effective and contextualized learning in a rich and attractive environment. Recognizing students’ emotions in a variety of situations can help both teacher and student develop skills that ensure a competent and coherent training process, positively influencing future patient care.

Although this is the first study to address the monitoring of emotions against unpleasant odors, the population, with 24 students, is a limiting factor. Considering that this is a comparative study on the effect of exposure to unpleasant odors in clinical simulation environments on the emotions of undergraduate nursing students, the sample was for convenience. Within the groups, students with and without experience in clinical practices were part of the study, which made it impossible to evaluate the relationship of emotions in these groups. In addition, there is a lack of studies on the topic addressed, which could be used to compare with the results found.

## Conclusion

This study concluded that 83,215 emotions were identified in the six points of the scenario, which demonstrates that clinical simulation can be an important tool in the training of nonverbal communication and emotions when facing unpleasant situations, such as the presence of bad odors. At the critical point of the proposed scenario with exposure to unpleasant odors, the IG presented the basic emotion of sadness and the CG, anger. The inclusion of unpleasant odors in the simulated scenarios can broaden the emotional development of health students. The results drive further studies in the area and showed that through clinical simulation it is possible to expand the learning of emotional aspects, increasing autonomy, coping with situations and student productivity.

The use of simulated practices that increasingly incorporate factors that mimic clinical practice and patient assessment are relevant tools for the development of clinical thinking and for the training of professionals. When such strategies are associated with the use of technologies and computational tools, such as those used here, they are relevant instruments in the self-knowledge and self-assessment of future professionals. The results and limitations found push further studies on the subject.
